# A Case of Diabetic Ketoacidosis Presented With Severe Rhabdomyolysis-Induced Acute Kidney Injury

**DOI:** 10.7759/cureus.38042

**Published:** 2023-04-24

**Authors:** Toka Amin, Mahmoud Nassar, Hazem Abosheaishaa, Amr Ali, Milana Zirkiyeva, Ricardo Lopez

**Affiliations:** 1 Internal Medicine, Icahn School of Medicine at Mount Sinai, Queens Hospital Center, New York, USA; 2 Gastroenterology, Cairo University, Cairo, EGY; 3 Pathology, Donald and Barbara Zucker School of Medicine at Hofstra/Northwell, New York, USA

**Keywords:** dialysis, muscle biopsy, diabetic ketoacidosis, acute kidney injury, rhabdomyolysis

## Abstract

We present a patient who presented with diabetic ketoacidosis and severe rhabdomyolysis-induced acute kidney injury. The patient developed generalized edema, nausea, and vomiting, and his kidney function deteriorated, necessitating renal replacement therapy, despite the successful treatment of his initial conditions. A comprehensive evaluation was conducted to determine the underlying cause of the severe rhabdomyolysis, including autoimmune myopathies, viral infections, and metabolic disorders. A muscle biopsy revealed necrosis and myophagocytosis but no significant inflammation or myositis. The patient's clinical and laboratory results improved with appropriate treatment, including temporary dialysis and erythropoietin therapy, and he was discharged to continue his rehabilitation with home health care.

## Introduction

Diabetic ketoacidosis (DKA) is a serious diabetes complication with relative insulin deficiency with less endogenous or exogenous administration, resulting in high blood sugar and ketone production. It can cause metabolic disturbances and organ failure or death. The most common precipitating factor is infection. Treatment entailed prompt insulin administration and correcting fluid and electrolyte imbalances to prevent complications such as cerebral edema and acute kidney injury [1].

Rhabdomyolysis is a clinical syndrome that releases myoglobin, creatine kinase, and other muscle breakdown products into the bloodstream. Various factors, such as trauma, drugs, infections, and metabolic disorders, can cause rhabdomyolysis, resulting in complications such as acute kidney injury, electrolyte imbalances, and metabolic acidosis. In this instance, the patient developed severe rhabdomyolysis, likely caused by hyperglycemia and dehydration associated with DKA and other factors. In this instance, the management of rhabdomyolysis included aggressive fluid resuscitation and electrolyte replacement, as well as monitoring for acute kidney injury and other complications [2].

## Case presentation

We present the case of a morbidly obese 33-year-old male with no significant past medical history in the past. He arrived at the emergency department with an altered mental status and red urine. The patient's blood pressure was 110/50 mmHg, his heart rate was 90 beats per minute, and his respiratory rate was 24 breaths per minute. The patient was lethargic and not disoriented to person, place, or time, but verbal stimuli elicited a response. Oral mucous membranes were dry, and generalized muscle tenderness was noted on the physical exam. A bedside point of care ultrasound (POCUS) revealed inferior vena cava less than 1.5 cm in diameter with near total collapse on inspiration, suggesting intravascular volume depletion and a hyperdynamic left ventricle. Routine labs were obtained at the time of presentation (table 1). The urine drug screen test was negative. A head computed tomography (CT) revealed no acute findings.

**Table 1:** Initial lab results BUN: Blood Urea Nitrogen; TSH: Thyroid stimulating Hormone; HbA1c: glycated hemoglobin test; eGFR: Estimated Glomerular Filtration Rate.

Laboratory test	Result	(reference range)
Hemoglobin	14 g/dL	(14-18 g/dL)
Leukocyte count	11.24 x10^3^/mcL	(4.8-10.8x10(3)/mcL)
Platelet count	296 x10^3^/mcL	(150-450x10(3)/mcL )
Sodium	161 mmol/L (measured) 180 mmol/L (corrected value for hyperglycemia)	(136-145 mmol/L)
Potassium	5.6 mmol/L	(3.5-5.1 mmol/L)
Chloride	102 mmol/L	(98-108 mmol/L)
Calcium	8.4 mg/dL	(8.6-10.3 mmol/L)
Phosphorus	2.8 mg/dL	(2.5-4.5 mg/dL)
BUN	86 mg/dL	(6-23 mg/dL)
Creatinine	5.02 mg/dL	(0.7-1.2 mg/dL)
eGFR	15 ml/min/1.73m2	(>=60 ml/min/1.73m2)
Serum glucose	1303 mg/dL	(74-110 mg/dL)
Measured HCO_3_	9 mmol/L	(22-29 mmol/L)
Anion gap	41 mEq/L	(8-16 mEq/L)
Beta-hydroxybutyrate	13.9 mmol/L	(ref. <=0.4mmol/L)
pH (venous)	7.17	(7.32-7.43)
PCO_2_ (venous)	23 mmHg	(41-54 mmHg)
PO_2_ (venous)	60 mmHg	(30-50 mmHg)
HCO_3_ (venous)	8 mmol/L	(22-29 mmol/L)
HbA1c	12.4%	(4-5.6%)
TSH	0.97	(0.27-4.2 uIU/mL)
Serum creatine kinase	>22000 U/L	(20-190 U/L)
Urine pH	6	(5-7.5)
Urine specific gravity	>1030	(1005-1030)
Urine ketones	15 mg/dL	(negative mg/dL)
Urine glucose	>1000 mg/dL	(negative mg/dL)
Urine protein	300 mg/dL	(negative mg/dL)
Urine blood	LARGE	(negative)
Urobilinogen	0.2 mg/dL	(0.2-1.0 mg/dL)
Urine RBCs	0-3/HPF	(0-3/HPF)
Urine WBCs	7-10/HPF	(0-4/HPF)
Urine bacteria	Moderate	(negative)
Urine yeast	Negative	(negative)
Urine nitrite	Negative	(negative)
Urine Leukocyte esterase	Negative	(negative)
Urine urate crystal amorphous	Few	(none)
Urine squamous epithelial cells	7-10/HPF	(0-4/HPF)
Urine hyaline casts	0-4/Ipf	(0-4/Ipf)
Urine pathologic casts	None	(None)

The patient was admitted to the intensive care unit (ICU) to treat DKA and acute kidney injury (AKI) caused by severe rhabdomyolysis. Over the following two days, the patient received 2 L of fluid bolus followed by continuous intravenous fluids with an average daily fluid intake of 5.5 L and average daily urine output of 2.6 L. This was accompanied by a downtrend in serum creatinine from 5.02 on admission to 1.69 on the third day. Insulin infusion continued until the anion gap closed and the DKA resolved. The hypernatremia gradually improved within 10 days of the diagnosis, and a normal serum sodium level was reached. Despite recovery from diabetic ketoacidosis and correction of hypernatremia, the patient developed generalized edema.

He continued to experience nausea and vomiting and remained lethargic with fluctuating mental status. Despite aggressive therapy with intravenous fluids, creatinine kinase (CK) remained elevated, and serum blood urea nitrogen (BUN) and creatinine levels trended again with a peak of 138 and 6.48, respectively. The patient had an average daily urine output of 2 L and net 1 to 4 L positive daily fluid shift. The urine protein creatinine ratio and the urea concentration in urine were noted to be 1.6 and 716 mg/dL, respectively. The patient developed nonhemolytic blood loss with a gradual hemoglobin drop to 6.6 g/dl with an elevated haptoglobin of 304 mg/dL and reticulocytes of 2.47%, which warranted a blood transfusion. Although serum CK decreased on the eleventh day to 5310 U/L, a subsequent rise of 11,371 U/L, the daily fluid retention, and ineffective diuresis prompted the start of renal replacement therapy with hemodialysis.

The workup of severe rhabdomyolysis and myositis has been done (Tables 2, 3). The right rectus femoris muscle biopsy revealed scattered atrophic and necrotic myofibers with segmental necrosis and myophagocytosis. However, there were no signs of significant inflammation, myositis, vasculitis, or evidence of group or selective atrophy, vacuolar changes, or dystrophic findings.

**Table 2:** Rhabdomyolysis workup TSH: Thyroid Stimulating Hormone; LDH: Lactate Dehydrogenase; TIBC: Total Iron Binding Capacity; C3: Complement 3; C4: Complement 4

Laboratory test	Results	(reference range)
TSH	0.97	(0.27-4.2 uIU/mL)
Iron	62 ug/dL	(45-165 ug/dL)
TIBC	276 ug/dL	(220-430 ug/dL)
Iron saturation	23%	(16-55%)
Ferritin	910 ng/mL	(30-400 ng/mL)
Reticulocyte count	2.47%	(0.5-1.5%)
Haptoglobin	304 mg/dL	(34-200 mg/dL)
Aldolase	66.9 U/L	(3.3-10.2 U/L)
LDH	592 U/L	(135-225 U/L)
Direct antiglobulin test	Negative	(negative)
C3	151 mg/dL	(81-157 mg/dL)
C4	39 mg/dL	(13-39 mg/dL)
Hemoglobin electrophoresis	Hemoglobin A 97.4% Hemoglobin A2 2.6%	(95.8-98%) (2-3.2%)

**Table 3:** Myositis-specific and associated antibodies ANA: Anti-nuclear antibody; Anti-dsDNA: Anti-double strand DNA; Anti-SRP antibody: Anti-signal recognition particle antibody; Anti-Mi-2 antibody: Anti-Dermatositis specific antibody; Anti-SCL 70: Anti-Scleroderma Anti-body; Anti-GBM: Anti-Glomerular Basement Membrane; p-ANCA, c-ANCA, atypical ANCA: perinuclear anti-neutrophil cytoplasmic antibodies, Cytoplasmic anti-neutrophil cytoplasmic antibodies, atypical anti-neutrophil cytoplasmic antibodies

Laboratory test	Result	(reference range)
Antisynthetase antibodies: JO-1, OJ, EJ, PL-7, PL-12	Negative	Negative
Anti-SRP antibody	Negative	Negative
Anti-Mi-2 antibody	Negative	Negative
Anti-MDA5 (CADM-140) antibody	Negative	Negative
Anti-TIF-1 gamma (P155/140) antibody	20 Units	(<20 Units)
Anti-NXP-2 (P140) antibody	Negative	Negative
Anti-SS-A-52 KD antibody	53 Units	(<20 Units)
Anti-U1-RNP	Negative	Negative
Anti U2 SNRNP	Negative	Negative
Anti Fibrillarin U3 -RNP	Negative	Negative
Anti PM/SCl 100	Negative	Negative
ANA	Negative	Negative
Anti-dsDNA	<12	(<=29 IU/mL)
Anti-smith antibodies	Negative	Negative
Anti-Scl 70	Negative	Negative
Anti-GBM	Negative	Negative
p-ANCA, c-ANCA, atypical ANCA	Negative	Negative
Anti-SS-A, Anti-SS-B	<0.2 AI	(<= 0.9 AI)
Cryoglobulins	Negative	Negative

The patient's clinical and laboratory results improved significantly with temporary dialysis. The kidney function improved, and the CK level decreased. Anemia improved with weekly Aranesp and ferrous sulfate. The patient was discharged to continue rehabilitation with home health care.

## Discussion

Rhabdomyolysis refers to the rapid breakdown of skeletal or striated muscle. Therefore, it is characterized by muscle fiber rupture and necrosis, which releases cellular products into the blood and extracellular space [3]. In the United States, approximately 26,000 cases of rhabdomyolysis require hospitalization per year [4]. Males, African Americans, people aged 10 to 60, and those with body mass indices greater than 40 kg/m^2^ appear to be more susceptible to the disease, which corresponds to our case sex, age, race, and BMI [5].

The etiology of rhabdomyolysis is classified into genetic or acquired; genetic causes include diseases of lipids or carbohydrate metabolisms like McArdle's disease and Tarui's disease, while acquired causes are further divided into traumatic including crush injuries, electrical injuries, compression, and vascular or orthopedic surgeries, and non-traumatic including vigorous exercises, seizures, sickle cell trait, exposure to extreme heat, malignant hyperthermia and neuroleptic malignant syndrome, alcohol, infections, electrolyte imbalance and drugs/toxins [6].

Depending on the degree and severity of muscle damage, rhabdomyolysis can present clinically in a wide range of ways, from an asymptomatic rise in serum levels of enzymes released from muscle cells, such as CK, LDH, and AST, to dire conditions like severe hypervolemia, metabolic acidosis, multiple electrolyte abnormalities, including hyperkalemia, hyperphosphatemia, and hypocalcemia, and acute kidney failure. Muscle pain, weakness, and dark urine are the "classic" triad of symptoms. However, only 10 % of patients report these symptoms [7].

Many case reports have linked hypernatremia and severe dehydration to rhabdomyolysis with a hyperosmolar state secondary to diabetes as the underlying mechanism [8-11]; however, Denman 2007 reported that hypernatremia alone might be a potential cause of rhabdomyolysis [12]. In 2014, Wenjing Li et al. reported two cases of rhabdomyolysis complicated by acute renal failure secondary to DKA and hyperosmolar hyperglycemia [13]. Recently, Burden et al. reported a case of West Nile virus-induced encephalitis and DKA complicated by rhabdomyolysis [14]. Al-Azzawi et al. concluded that patients with profound acidosis, urinary ketones in moderate to severe DKA, had a 6.98% chance of developing rhabdomyolysis; based on this evidence, every patient with severe DKA should be screened for rhabdomyolysis [15].

## Conclusions

This case report is the diagnostic and management challenges of severe rhabdomyolysis, which can result in various complications, including acute kidney injury, edema, and persistent muscle weakness. Despite extensive testing for autoimmune, viral, and metabolic disorders, the underlying cause of the rhabdomyolysis in this patient remained unclear. However, appropriate treatment with temporary dialysis and erythropoietin therapy led to significant clinical and laboratory improvement.

## References

[1] Kitabchi AE, Umpierrez GE, Miles JM, Fisher JN (2009). Hyperglycemic crises in adult patients with diabetes. Diabetes Care.

[2] Bosch X, Poch E, Grau JM (2009). Rhabdomyolysis and acute kidney injury. N Engl J Med.

[3] Hendgen-Cotta UB, Flögel U, Kelm M, Rassaf T (2010). Unmasking the Janus face of myoglobin in health and disease. J Exp Biol.

[4] Sauret JM, Marinides G, Wang GK (2002). Rhabdomyolysis. Am Fam Physician.

[5] Chavez LO, Leon M, Einav S, Varon J (2016). Beyond muscle destruction: a systematic review of rhabdomyolysis for clinical practice. Crit Care.

[6] Cabral BM, Edding SN, Portocarrero JP, Lerma EV (2020). Rhabdomyolysis. Dis Mon.

[7] Zutt R, van der Kooi AJ, Linthorst GE, Wanders RJ, de Visser M (2014). Rhabdomyolysis: review of the literature. Neuromuscul Disord.

[8] Tuller MA (1971). Acute myoglobinuria with or without drug usage. JAMA.

[9] Gangopadhyay KK, Ryder RE (2006). Nontraumatic rhabdomyolysis: an unusual complication of diabetic hyperosmolar nonketotic (HONK) state. J R Soc Med.

[10] Incecik F, Herguner MO, Yildizdas D, Ozcan K, Altunbasak S (2006). Rhabdomyolysis caused by hypernatremia. Indian J Pediatr.

[11] Acquarone N, Garibotto G, Pontremoli R, Gurreri G (1989). Hypernatremia associated with severe rhabdomyolysis. Nephron.

[12] Denman JP (2007). Hypernatraemia and rhabdomyolysis. Med J Aust.

[13] Li W, Gong C, Wu D, Liu M (2014). Two case reports of severe pediatric hyperosmolar hyperglycemia and diabetic ketoacidosis accompanied with rhabdomyolysis and acute renal failure. J Pediatr Endocrinol Metab.

[14] Burden Z, Fasen M, Judkins BL, Isache C (2019). A case of West Nile virus encephalitis accompanied by diabetic ketoacidosis and rhabdomyolysis. IDCases.

[15] Al-Azzawi OF, Razak MK, Al Hammady SJ (2019). Rhabdomyolysis; is it an overlooked DKA complication. Diabetes Metab Syndr.

